# Harm reduction in Asia and the Pacific: an evolving public health response

**DOI:** 10.1186/s12954-015-0074-x

**Published:** 2015-10-16

**Authors:** Nick Crofts, Tasnim Azim

**Affiliations:** Centre for Law Enforcement and Public Health, 309 George St, Doncaster, Vic 3108 Australia; icddr,b, GPO Box 128, Dhaka, 1000 Bangladesh

More than two decades ago, attention was called to the uncontrolled spread of injecting drug use in many Asian countries and the virtually unnoticed but rapid spread of HIV among those who were injecting [1]. It was pointed out that constantly changing trafficking routes from the Golden Triangle were exposing new populations to the use and subsequent injecting of heroin, that these populations were forming the fertile ground for explosive but largely silent epidemics of HIV and that there were structural and other reasons why these epidemics would prove difficult to control. It was called an evolving public health crisis; the authors concluded that

Vigorous efforts are urgently required at both a national and international level to raise awareness of the consequences of an uncontrolled epidemic of HIV among IDUs in Asian and other countries, and to support the implementation of policies which are likely to reduce the spread of HIV [1].

The initial challenge to the public health community was to gain acknowledgment of the existence of both epidemics—of drug injecting and of HIV among people who were injecting. Many original national medium-term AIDS plans from countries in the Asian region did not even mention injecting drug use as a component of the national epidemic, despite evidence from early in the course of the epidemic of explosive spread, high prevalences and the central role of epidemics of HIV among people who inject drugs (PWID) in driving national epidemics.

The second challenge was to gain recognition that these epidemics of HIV among and from PWID *mattered* and that it was necessary to control HIV spread among PWID to stop the national HIV epidemic. This challenge was increasingly taken up by the growing NGO sector, with harm reduction programs springing up in most countries in the region, most often in very adverse circumstances. In 1997, these programs came together at the International Harm Reduction Conference in Hobart to form the Asian Harm Reduction Network, which became the lead advocacy body for harm reduction responses in Asia.

These early harm reductionists also took on the next challenge, that of convincing authorities that something could be done to address and control these HIV epidemics and that it was worth doing. Official points of view, on the part of both national health and criminal justice sectors, were generally opposed to these positions: that there was nothing to be done about HIV among PWID (a view held for various and mostly spurious reasons) and that anyway, it did not matter because the epidemic would remain confined to populations of PWID, a group seen universally as socially undesirable, if not worse. Allied with the deeply felt desire to stamp out drug use, which was seen as a threat to Asian societies, this stance militated strongly against the adoption of effective measures—measures for the effectiveness and safety of which there was by then a large body of international evidence [2].

Increased attention to the issue followed high-profile epidemics of HIV among and increasingly *from* PWID, which occurred around the Burmese borders, following heroin trafficking routes [3], especially in the southwest of China, in Ruili county and in the northeast of India, in Manipur State. By 2000, one study found that of young PWID in Manipur, 75 % were infected with HIV and 98 % had been exposed to the hepatitis C virus (HCV)—this despite the fact that Manipur was the first jurisdiction in the Asian regions to explicitly adopt harm reduction as policy [4]. The scale of the response was manifestly insufficient to match the scale of the epidemic, and in most places, what response there was came well after the epidemic was established. For instance, Bangladesh, which responded early, has maintained low HIV prevalence among PWID [5]; Pakistan, where the response was much later, has seen continuing epidemics [6]. It is a truism in the field of control of blood-borne viral infections that much greater prevention efforts are required when prevalences are high [7].

The evolution of drug production and consumption patterns, of social and geographic focuses of drug use and injection, and of viral epidemics associated with drug use has been rapid, constant and varied. New populations exposed by new trafficking routes and methods; new vulnerabilities created by social and economic development, with the appearance of irreversible structural unemployment associated with urbanisation; and new drugs appealing to hitherto uninvolved segments of society—all these factors have contributed to a constantly shifting and complex set of challenges to HIV control. The increasing use of amphetamines, originally largely occupational use in the region but rapidly becoming recreational among large communities of young people, has been described as a ‘flood’ across the region [8]. Increasing crossover of risks—especially between injecting drug use and sex work—and continued incarceration and reincarceration of these populations have amplified spread of HIV and ensured it has affected whole communities—*not* just those who use drugs.

Has the public health response, in the form of harm reduction interventions, kept pace with these changes and reached the scale necessary to stop HIV among PWID in Asia? Certainly, there is an increasing number of programs providing sterile needles and syringes to an increasing number of clients [9] and an increasing number of countries are implementing opiate substitution programs, led by a startling *volte face* by China in relation to methadone maintenance in the early part of this century [10, 11]. But is it enough?

And beyond HIV, have harm reduction policies and programs brought substantial gains in access to effective drug treatment, in recognition of the human rights of people who use drugs and in elimination of prejudice, stigma and discrimination against people who use drugs? And where there have been successes, are they integrated into social systems and structures so as to become sustainable [12]?

This special issue of the Harm Reduction Journal brings together current research evidence to address these questions.

Harm reduction responses have indeed grown in the region over the last two decades, but what has been achieved in most countries has been the introduction of small programs reaching few people in need. Katie Stone’s report from the HR Global State of Harm Reduction survey in 2014 illustrates this: of 23 countries in south and SE Asia, 17 countries have needle and syringe program (NSP) provision and 15 opioid substitution therapy (OST); coverage, however, remained low, with the numbers reached with OST in the few thousands and less than 20 % of those infected with HIV on antiretroviral treatment (ART) [9]. An example is Pakistan where HIV prevalence in this population is above 40 % in several cities, including Faisalabad (52.5 %), D.G. Khan (49.6 %), Gujrat (46.2 %), Karachi (42.2 %) and Sargodha (40.6 %) [6]. Current coverage of the needle and syringe program, HIV testing and counselling and ART among PWID remains insufficient: of an estimated 430,000 PWID in Pakistan, around one tenth are registered with harm reduction programs and less than 10 % of those in need of ART are receiving treatment [6].

As well as inadequate attempts to address these needs, there are huge gaps yet to be addressed: most PWID are infected with HCV, for instance, but treatment remains beyond the reach of virtually all. In Kabul, a conflict-affected area, Todd et al. show a high HCV incidence and high numbers of reported deaths among male PWID despite relatively consistent levels of harm reduction program use [13]. The case is similar for those PWID with combined TB and HIV infection, for very few of whom is there available treatment [14].

Although the needle syringe program has been adopted in many countries in the region, sharing of used needles syringes continues to remain an issue [10]. WHO recommends the use of low dead space syringes (LDSS) with permanently attached needles in NSPs as these are better at preventing HIV and HCV transmission [15]. However, the acceptability of LDSS by PWID has been problematic as shown in the qualitative study conducted with PWID in Tajikistan by Zule et al., which shows that the size of the syringe as well as being able to detach the needle from the syringe are important aspects to injection practices [16]. Such barriers may be overcome through social marketing interventions as demonstrated in the paper by Huong et al., where such an intervention in Vietnam was found to play an important role in widening access to and the use of LDSS for PWID [17].

HIV in PWID in the Asia Pacific region is a mixed picture with declining trends but emerging pockets [18]. The need to better understand the epidemic is highlighted in the paper by Todd et al. that summarises the discussions of a consultative meeting reviewing what is known, what needs to be known and how to move forward so that the universal goal of 90-90-90 can be achieved by engaging communities for reaching those most hidden, enhancing HTC coverage and ensuring adherence for ART [19]. Similarly, from the Pacific region where information is limited on drug use behaviours, Power et al. stress the need for a better understanding of the local situation in order to ensure more appropriate programming—especially recognising the diversity of situations, summarised in the phrase ‘know your epidemic’ [20].

It is encouraging to see that OST is gaining acceptance in most countries in the region although coverage is far from adequate. Three papers presented in this issue discuss beneficial effects of methadone maintenance treatment (MMT) on clients as well as factors that could improve uptake of methadone. Hoang et al. show that in Vietnam, a successful pilot of MMT has led to considerable expansion; they have gone further to show that being on ART or being co-infected with TB may act as barriers to adherence, factors which need to be addressed in the MMT programs [21]. Similar findings have been reported from Taiwan by Chang et al. where the quality of life of MMT clients improved but co-infections such as with HCV were predictors of non-adherence [22]. In Hong Kong, where MMT has been in place for about 40 years, Kwan et al. have analysed records of MMT clients since 2008 and shown that optimal frequency of attendance at MMT program is dependent on an adequate dosage and that the connectivity of methadone users among themselves could impact harm reduction intensity [23].

There is a cruel irony in the fact that methadone is becoming established just as Amphetamine-type stimulants (ATS) take over as dominant drug [8]. The four papers in this issue on women who use drugs all report ATS as the dominant drug used.

A recently published meta-analysis on the number of females who inject drugs (FWID) showed that of people who inject drugs, around 21.5 % are women, which would correspond to approximately 3.5 million FWID globally [24, 25]. Women who use drugs are highly stigmatised and suffer from multiple risks related to drug use, unsafe sex and violence [26]. In this issue, four papers present different dimensions of women using drugs from China, Vietnam, Cambodia and Malaysia. In Yunnan, China, Zhang et al. highlight the special needs of young women who often sell sex to support their ATS use and the risky sexual practices associated with ATS alongside a lack of knowledge and understanding of those risks and access to services [27]. Similarly, the paper from Vietnam by Morrow et al. reveals the vulnerabilities of women who use drugs in Hanoi and Ho Chi Min City both in terms of their individual behaviours (unsafe injecting and unsafe sex) as well as high levels of stigma [28]. The study from Cambodia by Dixon et al. on female entertainment sex workers (FESW) presents a different dimension of women’s vulnerabilities associated with ATS use, where ATS is used for occupational performance—to stay awake longer and to work more hours, enabling FESW to see more clients and also in some cases to be “happy” and to forget about their problems [29]. Present harm reduction services are geared towards individual behaviour change but fail to recognise these structural issues that make it difficult for individuals to adopt safer behaviours, such as condom negotiation when both FESW and their clients are using ATS. The role of familial instability as a gateway to drug use is presented in the qualitative study from Malaysia by Rahman et al. on a small group of women who mainly smoke heroin or ATS; with a lack of appropriate services and interventions, these women were condemned to recreate the unstable backgrounds from which they came [30].

As has been found in other places, the role of the at-risk communities is a critical one in building successful responses to HIV epidemics. Le et al. highlight the efforts that are underway in Vietnam for CBOs and the government to work together, as there is increasing recognition that community-based organisations (CBOs) have greater access to the marginalised communities of people who use drugs and/or sell sex [31]. Building partnerships with police, in particular, is critically important for effective responses [32]. And it is becoming very clearly recognised that unidimensional law enforcement approaches to illicit drug use, and a reliance on arbitrary detention, based on administrative regulation and masquerading as ‘drug treatment’, is not only ineffective but also extremely deleterious, both to the individual and to their communities [33], that the death penalty for drug-related offences that continues to be used is unconscionable [34].

What is the future of harm reduction in the Asian and Pacific regions in the face of shrinking resources and the rise of competing priorities?

A very real and ever-present danger is that with apparent control of the epidemic, attention will shift away from PWID and gains in human rights, rule of law and effective approaches to treatment achieved at great cost will be wound back. A further irony is that without an HIV epidemic, it is very unlikely that the human rights of people who inject drugs would ever have become a topic for international discussion and funding. If the HIV epidemic is perceived to be over, will countries return to their previous unconcern and inhumanity towards these people? Or have we actually learnt something from the struggle against HIV?
